# Illuminating necrosis: From mechanistic exploration to preclinical application using fluorescence molecular imaging with indocyanine green

**DOI:** 10.1038/srep21013

**Published:** 2016-02-11

**Authors:** Cheng Fang, Kun Wang, Chaoting Zeng, Chongwei Chi, Wenting Shang, Jinzuo Ye, Yamin Mao, Yingfang Fan, Jian Yang, Nan Xiang, Ning Zeng, Wen Zhu, Chihua Fang, Jie Tian

**Affiliations:** 1 Department of Hepatobiliary Surgery, Zhujiang Hospital, Southern Medical University, Guangzhou 510280, China; 2 Key Laboratory of Molecular Imaging, Institute of Automation, Chinese Academy of Sciences, Beijing 100190, China; 3 Beijing Key Laboratory of Molecular Imaging, Beijing 100190, China

## Abstract

Tissue necrosis commonly accompanies the development of a wide range of serious diseases. Therefore, highly sensitive detection and precise boundary delineation of necrotic tissue via effective imaging techniques are crucial for clinical treatments; however, no imaging modalities have achieved satisfactory results to date. Although fluorescence molecular imaging (FMI) shows potential in this regard, no effective necrosis-avid fluorescent probe has been developed for clinical applications. Here, we demonstrate that indocyanine green (ICG) can achieve high avidity of necrotic tissue owing to its interaction with lipoprotein (LP) and phospholipids. The mechanism was explored at the cellular and molecular levels through a series of *in vitro* studies. Detection of necrotic tissue and real-time image-guided surgery were successfully achieved in different organs of different animal models with the help of FMI using in house-designed imaging devices. The results indicated that necrotic tissue with a 0.6 mm diameter could be effectively detected with precise boundary definition. We believe that the new discovery and the associated imaging techniques will improve personalized and precise surgery in the near future.

Necrosis is a common indicator of the occurrence and development of various diseases and is also one of the major risk factors for accelerated deterioration of diseases. If proper diagnosis and intervention are not achieved in a timely manner, the evolution of necrosis in vital organs may lead to fatal outcomes^1^,^2^,^3^,^4^. Therefore, highly sensitive detection and precise boundary delineation of necrotic lesions are crucial for clinical diagnosis and surgical treatment in order to achieve complete removal of the necrotic tissue as well as to minimize the loss of healthy tissue^5^,^6^. Furthermore, these techniques are also extremely valuable for the prognosis of malignant tumours and evaluation of therapeutic effects^7^. Therefore, different imaging strategies and contrast agents or probes have been proposed to detect necrosis^8^,^9^,^10^,^11^.

Clinically applied imaging modalities such as ultrasound, computed tomography (CT), magnetic resonance imaging (MRI), and positron emission tomography (PET) rely on either the perfusion of contrast agents in normal tissues or necrosis-avid imaging probes to indirectly or directly detect necrotic lesions^8^,^9^,^10^,^12^. The indirect approaches have the disadvantages of inaccurate estimation of the necrotic margin and a short observation window^7^,^9^. For example, indocyanine green (ICG) has been used for intraoperative fluorescence angiography to detect tissue ischemia^4^ based on its insufficient delivery to the ischemic area in the observation window (several minutes) after intravenous (IV) administration. However, this indirect approach cannot distinguish between the necrotic tissue and reversible ischemic tissue^13^. It is also less sensitive in detecting small necrotic or ischemic tissue due to the optical scattering effect from the much larger area of normal tissues. Furthermore, the short observation window (several minutes) limits its application for observations of the long-term dynamics during surgery^14^. Applying necrosis-avid probes for direct imaging, such as radioisotope-labelled hypericin, can offer better overall performance^10^; however, due to the limitation of conventional imaging modalities, it is challenging to achieve both high sensitivity for small necrotic lesion detection and precise definition of the necrotic boundary^15^. Fluorescence molecular imaging (FMI) and associated intraoperative image-guided surgery have proven to be effective with respect to both sensitivity and boundary definition, demonstrating potential preclinical and clinical applications^16^,^17^,^18^,^19^; however, these novel imaging techniques have not yet been applied for necrosis diagnosis and clinical treatment, mainly because of the lack of a suitable fluorescent probe.

The typical method for developing disease-targeted fluorescent probes requires covalent conjugation of a targeting component (for example, a peptide or antibody) and a near-infrared (NIR) fluorophore^20^,^21^. Although this strategy works well in preclinical applications, the synthetic conjugates are relatively large molecules and it is thus challenging to obtain immediate clinical translation due to the long time required for obtaining Food and Drug Administration (FDA) approval^22^,^23^. Therefore, there is an urgent demand for an ideal fluorescent probe (i.e., a small molecule with superb necrosis specificity) that already holds FDA approval for clinical applications. This would potentially enable the use of optical imaging techniques for the clinical diagnosis and treatment of necrosis-associated diseases with high sensitivity and high superficial resolution.

Here, we demonstrate that ICG, an FDA-approved NIR fluorescent dye^24^, has previously undiscovered ability to selectively bind to necrotic cells because of its interaction with lipoprotein (LP) and phospholipids, which is driven by its inherent chemical structure^25^. We explored the mechanism through a series of *in vitro*, *in vivo*, and *ex vivo* experiments based on previous studies^26^,^27^,^28^, in which comprehensive experimental data indicated that ICG binds to LP in the human blood circulation. Another report suggested a binding effect between ICG and human serum albumin (HSA)^29^, but this study was performed *in vitro* with ICG in an HSA solution rather than in a live blood circulation system. Therefore, our mechanism exploration focused on whether the bound ICG–LP molecules in living organisms exhibit necrosis avidity following IV injection of ICG.

We also investigated an improved ICG administration strategy to obtain a better signal-to-background ratio (SBR, the ratio of optical efficiency between the necrotic lesion and normal tissue). Furthermore, FMI and real-time image-guided surgery were applied to different animal models of necrosis-associated diseases using an in house-modified fluorescence microscope, which demonstrated the high sensitivity and accurate necrotic boundary delineation ability of this novel imaging technique. We believe that this important discovery and its related optical imaging techniques have great potential for clinical translation in a wide range of necrosis-associated diseases.

## Results

### Preliminary mechanistic exploration of the necrosis avidity of ICG

**Figure 1:**
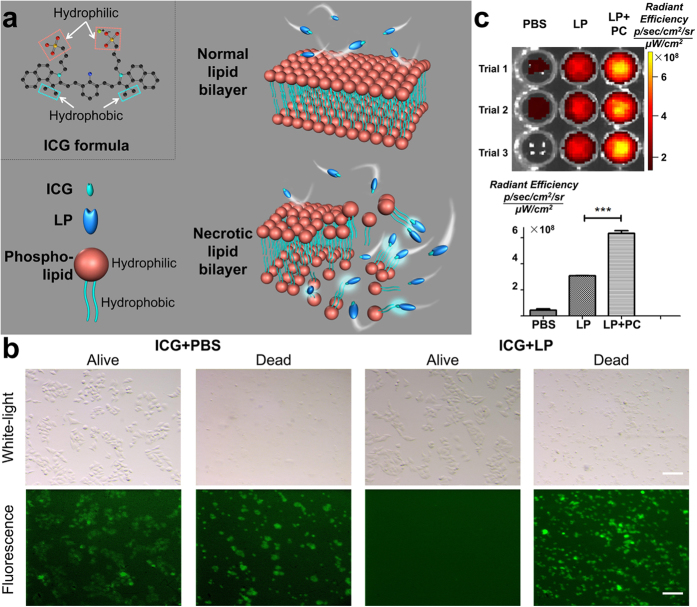
Schematic illustration of the mechanism of ICG to target necrotic cells. (**a**) The molecular structure of ICG (ICG formula) demonstrates its amphiphilic properties. For normal cells (normal lipid bilayer), the ICG-lipoprotein (LP) complex was not able to stick on the surface of the lipid bilayer, because the hydrophilic end of ICG was shielded by LP. For necrotic cells (necrotic lipid bilayer), the ruptured lipid bilayer was bound with ICG-LP due to exposure of the hydrophobic tails of phospholipids. (**b**) Free ICG molecules (ICG dissolved in PBS) were bound with both viable and necrotic cells (left two columns). ICG-LP molecules (ICG dissolved in fetal bovine serum) were bound with necrotic cells (fourth column), but were easily washed out from viable cells (third column). Scale bar, 30 μm. (**c**) Compared to ICG + serum solution (second column), the mixture of ICG + serum and PC, (third column) showed a significantly stronger fluorescence signal. The solution of ICG and PBS was used as reference (first column). The bottom bar graph illustrates the quantitative analysis. ***P < 0.001.

The molecular structure of ICG clearly demonstrates amphiphilic properties (Fig. 1a, ICG formula). The ICG-LP complex is too large (7–20 nm)^26^ to penetrate normal blood vessel walls, unless vascular permeability increases due to certain disorders such as a malignant tumour, inflammation, or trauma^26^,^30^. However, we hypothesized that even if the ICG-LP complex reaches the interstitial space, it will neither be actively transported into living cells^31^ nor attached to any molecules on the normal cell membrane surface (Fig. 1a, normal lipid bilayer), except for hepatocytes, which are responsible for quickly excreting ICG into the biliary system and gut without the enterohepatic circulation^30^,^31^. Furthermore, the metabolism and pharmacokinetics of ICG-LP in the lesion space of necrotic cells are completely different from those at other sites (Fig. 1a, necrotic lipid bilayer). Since LP shields the hydrophilic end of ICG, ICG-LP shows enhanced affinity for hydrophobic groups. Moreover, loss of cell membrane integrity due to necrosis exposes the hydrophobic tails of phospholipids, and thus ICG-LP might show distinct affinity for the phospholipids from the ruptured lipid bilayer.

We here preliminarily verified our mechanistic hypothesis of the necrosis avidity of ICG using a series of experiments at the cellular and molecular levels. *In cellulo* white-light and fluorescence images demonstrated that free ICG molecules dissolved in phosphate-buffered saline (PBS) bound to both viable and necrotic cells (4T1-fluc, luciferase-expressing mouse adenocarcinoma cells, Fig. 1b, ICG + PBS), whereas ICG-LP (ICG dissolved in foetal bovine serum) only showed affinity for necrotic cells and was completely washed out from viable cells (Fig. 1b, ICG + LP). This suggested that once ICG was IV-injected into the *in vivo* circulation system, the naturally generated ICG-LP complex would show higher accumulation in necrotic tissues.

*In vitro* quantitative comparison of the fluorescence efficiency among ICG with PBS (baseline), ICG with LP, and ICG with LP and phosphatidylcholine (PC) was performed in 96-well plates (Fig. 1C). Significant signal enhancement (105.84% increase, P < 0.001) was observed in the ICG + LP + PC group compared to the ICG + LP group, indicating that the interaction between the ICG-LP complex and PC significantly enhanced the optical signal. PC is the most dominant phospholipid in the cell membrane and is abundant in necrotic tissues^32^,^33^. This suggested that once ICG-LP bound to necrotic cells, significant optical contrast between necrosis and its surroundings could be observed. The fact that optical signal enhancement of ICG-LP was only observed in necrotic cell debris (Fig. 1b, ICG + LP) verified our hypothesis of how ICG-LP interacts with the ruptured lipid bilayer. Although this exploration remains preliminary, it has improved our understanding of the underlying mechanism.

### *In vivo* and *ex vivo* verifications

**Figure 2:**
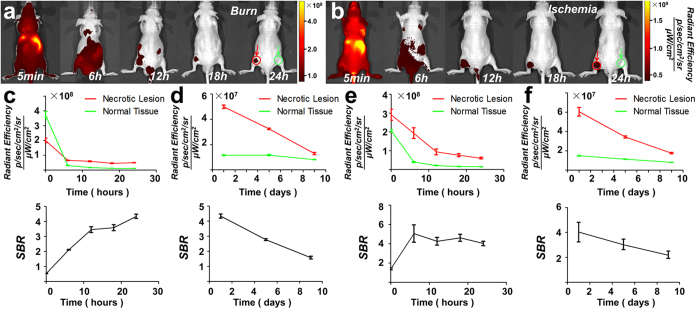
*In vivo* FMI of necrotic tissues in different mouse models. (**a**,**b**) For both the burn and muscle ischemia necrosis mouse models, FMI could indicate necrotic lesions 12 h after IV injection of ICG. The necrotic tissue and corresponding normal tissue are indicated by the red and green arrows, respectively. (**c**,**d**) For the burn necrosis mouse model, the optical efficiency profiles of the necrotic lesion and the normal tissue at 24 h and 9 days post-injection are illustrated in the upper graphs of (**c**,**d**), respectively. The SBR; ratio of optical efficiency between necrotic and normal tissues) profiles for 24 h and 9 days are illustrated in the bottom graphs of (**c**,**d**), respectively. On the ninth day, the average SBR was 1.58 ± 0.23 for burn lesions. (**e**,**f**) For the ischemia necrosis mouse model, the quantitative analysis of the optical efficiency and SBR at 24 h and 9 days post-injection are illustrated in the same layout as shown in (**c**,**d**). On the ninth day, the average SBR was 2.17 ± 0.22 for ischemia lesions. Both mouse models suggested the necrosis binding of ICG with a long observation window.

For further confirmation, a series of *in vivo* and *ex vivo* animal experiments were conducted. ICG solution (0.1 ml, 0.5 mg/kg body weight; a FDA-approved dose^24^) was IV-injected into nude mouse models of burn-induced and muscle ischemia-reperfusion-induced^34^ hind limb necrosis (n = 6 per model). Fluorescent images were acquired over 24 h at 5 time points (Fig. 2a,b). Necrotic lesions showed clear optical contrast with the surrounding tissue at 12 h post-injection in both models. Although the profiles of optical efficiency and the SBR of necrotic lesions differed in the two mouse models (burn: Fig. 2c; ischemia: Fig. 2e), the necrosis avidity of ICG-LP was clearly demonstrated in both cases. The *in vivo* experiments also proved that ICG could achieve a good selective necrosis-binding effect, even if the causes of necrosis are diverse.

Quantitative measurements of optical efficiency and SBR were also performed on the fifth and ninth days, and similar profiles were observed for the two models (burn: Fig. 2d; ischemia: Fig. 2f), indicating that the washing out of ICG from necrotic lesions was much slower than that from normal tissues. This suggested a long observation window up to several days with just a single ICG administration. In addition, since the SBR consistently decreased at 24 h after the injection in both mouse models, we considered 24 h post-injection as the standard *in vivo* observation time point for subsequent experiments.

**Figure 3:**
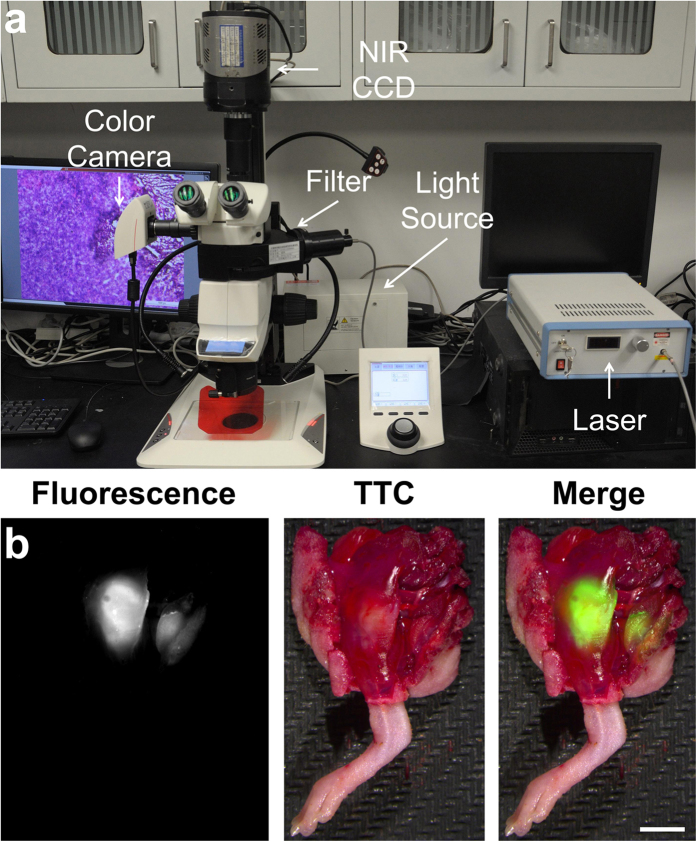
The in house-modified fluorescence microscope system and *ex vivo* verification. (**a**) A conventional microscope (Leica M205 FA, LEICA, Germany) was coupled with a low-temperature NIR CCD (PIXIS CCD, Princeton Instruments) and a color camera. A laser generator (MW-GX-785/2W, Changchun FS-Optics technology Co., Ltd., China) was coupled with an optical filtering module embedded in the microscope through an optical fiber, so that the excitation light with a selectable wavelength could be transmitted to an imaging target. The emission was also filtered by the filtering module and then captured by the NIR CCD. The light source provided white-light illumination for the color camera to acquire white-light images. The system can provide 0.5–160× magnification. (**b**) On the ninth day after ICG injection, the fluorescent image shows two ICG-accumulated areas in the resected mouse hind limb. After TTC staining of the entire limb, the ischemia-reperfusion-caused necrotic tissue locations were visible (white areas), which are the same sites where the ICG accumulated. Scale bar, 5 mm.

After the *in vivo* verification, the ischemic necrosis mice were then sacrificed for 2,3,5-triphenyltetrazolium chloride (TTC) staining^35^, and *ex vivo* fluorescent images were acquired using an in house-modified fluorescence microscope system (Fig. 3a). The system integrates a conventional microscope, a low-temperature NIR CCD, a colour camera, and a laser generator to achieve real-time fluorescence imaging. Since the *ex vivo* TTC staining does not compromise the ICG distribution, they can corroborate each other for necrosis detection. For all of the ischemia-reperfusion-caused necrosis specimens on the ninth day after ICG injection, the high optical contrast areas in the fluorescent and merged images were perfectly matched with the white tissue areas (necrotic tissues) in TTC staining (Fig. 3b). These results confirmed our findings.

### Improved ICG administration strategy to enhance the SBR

**Figure 4:**
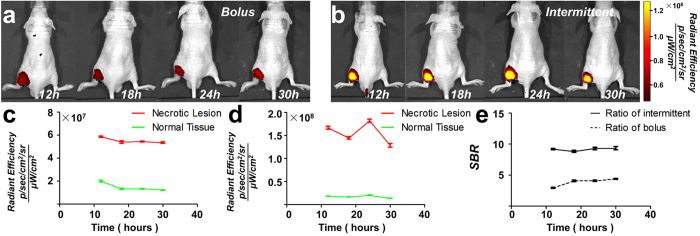
*In vivo* comparison of different ICG administration strategies. (**a**) ICG (2.0 mg/kg) was intravenously bolus-injected into the mouse, and FMI was performed at 4 time points. (**b**) The same dose of ICG was intermittently injected into the same mouse model. FMI captured an obviously stronger optical signal of the necrotic lesion. (**c**,**d**) Quantitative analysis confirmed that both injection strategies provided higher optical efficiency in necrotic tissues in comparison with normal tissues. (**e**) Comparison of the SBR further indicated that the optical signal of necrosis was at least 2-fold greater with intermittent injection than that obtained with bolus injection at each observation point.

The half-life of ICG in the blood circulation is only 2–4 min^30^, which restricts the delivery of ICG-LP into necrotic tissues. We therefore sought to determine whether the SBR could be increased using intermittent injections instead of a single IV bolus of ICG with the same dose. Using the burn-induced hind limb necrosis mouse model, the control group received a bolus IV injection of 0.1 ml ICG solution (2.0 mg/kg, which is the dose approved by the FDA^24^), and the experimental group received 4 intermittent injections of 0.1 ml ICG solution (0.5 mg/kg) at 3-h intervals. FMI was performed at 4 time points from 12 to 30 h post-injection (Fig. 4A,B). The optical efficiency of necrotic lesions was significantly larger than that of normal tissues at each time point in both groups (P < 0.001, Fig. 4c,d), which was consistent with our previous *in vivo* verifications (Fig. 2). However, at each time point, the SBR of the experimental group was at least 2-fold greater than that of the control (P < 0.001, Fig. 4e); in particular, at 24 h, the SBR of the experimental group was 9.28 ± 0.29. These results indicated that intermittent injections improved ICG-LP delivery to necrotic lesions. Therefore, the improved ICG administration strategy could significantly enhance the optical signal intensity of necrotic lesions without requiring an increase in the injection dose.

### Necrosis detection for various animal disease models

Necrosis is a common indicator for the development of many serious diseases. The discovery of the necrosis avidity of ICG-LP opened the gate for employing FMI to diagnose necrosis-associated diseases. Therefore, we applied this imaging technique to various disease mouse and rat models such as spontaneous necrosis of 4T1 breast cancer, brain necrosis, hepatic injury, and gastric ulcer to evaluate its accuracy for necrosis boundary definition and sensitivity to small-lesion detection.

**Figure 5:**
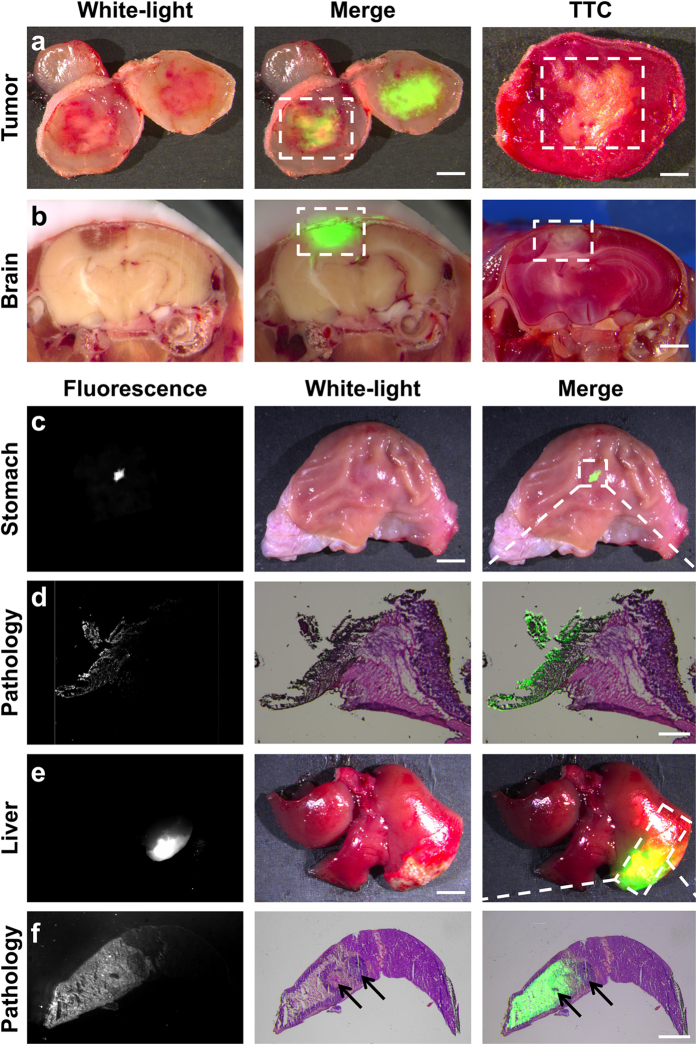
Definition of the necrosis boundary and small-lesion detection using FMI. (**a**,**b**) The white-light and merged images indicate the same location of the necrotic tissue inside the 4T1 tumor and brain. The contour of the fluorescent region matched the contour of the white region after TTC staining (dashed squares in the merge and TTC images). Scale bars: (**a**) Merge: 3 mm, TTC: 2 mm, (**b**) 2 mm. (**c**) The fluorescent image shows a very small necrotic lesion (0.6 mm) in the gastric ulcer, which is unrecognizable in the white-light image. Scale bar, 2 mm. (**d**) Boundary delineation via ICG signals showed a perfect match with hematoxylin and eosin pathology at the microscopic level. Scale bar, 50 μm. (**e**) Compared with the white-light image, the fluorescent image showed a larger area of necrotic tissue in the liver. Scale bar, 4 mm. (**f**) Pathologic images confirmed more accurate boundary delineation with fluorescence. The black arrows indicate the small areas of normal tissues embedded inside the necrotic tissues, which were successfully delineated. Scale bar, 2 mm.

The comparison between fluorescent images and TTC staining of the excised tumour and brain showed that the location and contour of the illuminated areas matched the necrotic lesions perfectly (Fig. 5a,b). This implies the potential of applying FMI with ICG for monitoring necrosis-associated oncotherapy and brain diseases.

Comparison of hematoxylin and eosin staining and fluorescent images of stomach and liver specimens at the microscopic level showed that the boundary of the necrotic area was precisely delineated by the contrast of the fluorescent signals (Fig. 5c–f). In particular, in the liver specimen, the tiny normal tissues embedded in the necrotic areas could be distinguished by the optical images (Fig. 5f, black arrows), indicating the high specificity of ICG for necrosis. Moreover, due to the inherent high sensitivity of FMI, tiny foci of necrosis could also be imaged. The smallest necrosis in the ulcer that was successfully detected was 0.6 mm in diameter (Fig. 5c,d; average ulcer size: 1.0 ± 0.3 mm, n = 6), indicating the potential of this technique for applications requiring high detection sensitivity.

### Preclinical translation of intraoperative image-guided surgery

Recently, real-time intraoperative image-guided surgery with FMI has been applied for sentinel lymph node biopsy, and ovarian and liver cancer resection^22^,^36^,^37^, which has revealed superior sensitivity and precise boundary definition ability compared to conventional methods. Therefore, we developed an in house-modified fluorescent microscopic system (Fig. 3a) that can be employed for real-time image-guided surgical navigation. Then, we applied the system and ICG administration to preclinical cases for feasibility assessment of the objective removal of necrotic tissues. The goal was to evaluate whether this imaging technique was valuable for clinical translation to assist surgeons.

**Figure 6:**
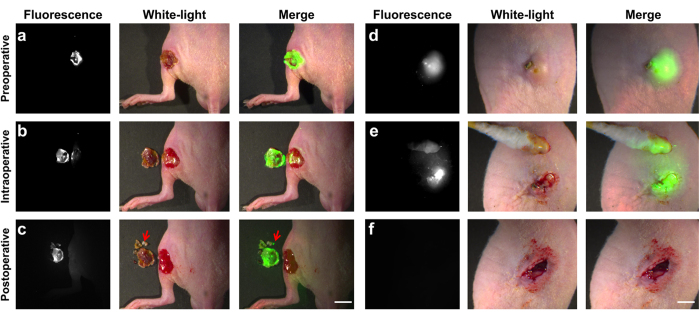
The intraoperative image-guided surgery of escharectomy and removal of a bacterial abscess. (**a**) The fluorescent image confirmed the location of the burn lesion before the escharectomy. (**b**) During the peeling of the eschar, fluorescent images were acquired to evaluate the residuals in a step-by-step manner. (**c**) The fluorescent image showed that all of the eschar was removed from the mouse. The resected tissues were placed beside the surgical area, which had a higher ICG concentration. The size of the smallest piece (red arrow) was only around 1 mm. Scale bar, 5 mm. (**d**) The fluorescent image confirmed the location of the abscess. (**e**) After abscess incision, the yellow pus was wiped away with a sterile cotton swab, and fluorescent images were acquired to evaluate the residuals in a step-by-step manner. (**f**) When all of the pus was removed, there was no fluorescent signal emitted from the lesion. Scale bar, 5 mm.

For the escharectomy of a burn (coagulative necrosis) in living mouse models, the fluorescent images provided accurate preoperative localization (Fig. 6a) and objective residue evaluation during the step-by-step resection process (Fig. 6b). Because of the high sensitivity and accuracy of this technique, complete removal of the necrotic tissue was achieved in all cases (n = 3) with maximum protection of the surrounding normal tissues (Fig. 6c). Even for a residue that was 1 mm in size, this optical technique could still be used as a guide for the surgeon to achieve accurate escharectomy (Fig. 6c, red arrow).

For the removal of a bacterial abscess (liquefactive necrosis) in living mouse models, even when the morphology of the necrotic tissue was continuously changing, the real-time fluorescent images could still offer objective intraoperative navigation and residue detection (Fig. 6d). In particular, when the majority of the abscess was removed, it was very difficult to estimate whether there was residue present with the naked eye (Fig. 6e, white light), whereas the surgeon could easily obtain valuable information through the guidance of FMI (Fig. 6f).

## Discussion

ICG is a small molecule that has been used in clinical settings for more than 40 years^30^. It was generally considered to be a non-specific NIR fluorescent dye. The discovery that ICG selectively binds to necrotic tissues and the preliminary explanation of the underlying mechanism via interaction between the ICG-LP complex and phospholipids may shift conventional awareness of the medical benefits associated with the use of ICG. This opens the door for the potential application of ICG to immediate medical translation.

Kokudo *et al*.^37^ first reported the phenomenon of ICG retention in hepatocellular carcinoma (HCC) in 2009, and immediately translated it clinically during hepatectomy for detection of superficial small tumour foci and metastatic tumours located in the liver^38^. However, the mechanism of the retention of ICG in liver cancer tissue and the necrosis affinity of ICG are completely different. The general mechanism of liver cancer detection using ICG is that the HCC cells sustain the function of normal liver cells to actively uptake ICG, whereas they lose the function of ICG excretion. This results in the retention of ICG in HCC cells and the consequent fluorescence contrast between tumours and normal liver tissue. Our discovery of the necrosis avidity of ICG is not limited in the application of liver tissue necrosis. It has a universal application in all necrosis-associated disorders in any tissue or organs. The discovery of the liver tumour detection ability of ICG based on this mechanism has already been widely exploited in clinical practice to benefit many patients. We believe that our discovery will also quickly be translated into clinical practice.

Several studies have demonstrated the use of ICG for identifying different malignant tumours such as pulmonary nodules, peritoneal ovarian cancer nodules, and 4T1 breast tumors^39^,^40^,^41^. In these studies, retention of ICG in tumour lesions was observed 24 h after the IV injection. The findings of a previous report that ICG could not be actively transported into living cells except liver cells or liver cancer cells^31^, as well as the result of our 4T1 tumour study that ICG actually accumulated in the necrotic part inside the tumour (Fig. 5a) suggest that the same phenomenon of the necrosis avidity of ICG occurred inside the tumours of previous studies with spontaneous or therapy-induced necrosis, although the underlying mechanism was not explored in these studies^39^,^40^,^41^. Another study demonstrated the use of ICG for atherosclerotic plaque imaging^42^. Although the phenomenon of ICG targeting to the plaque was observed, these authors also did not reveal the underlying mechanism, which is likely to be the binding of ICG-LP to the phospholipids of the necrotic cells or the deposited sediments inside the plaque; thus, these authors did not extend the application of ICG to other necrosis-associated disorders. Nevertheless, the essential finding of all these studies indicates the broadness of the potential clinical translations in using FMI with ICG.

In addition, we noticed that some necrosis avid materials are also apoptosis-avid^15^. Typically, the late stage of apoptosis is characterized according to the expression of phagocytotic molecules such as phosphatidylserine (PS) on the cell surface. ICG-LP is PC-avid and might also be PS-avid as well. However, the present mechanism exploration study showed that ICG-LP has affinity for the hydrophobic groups of PC. Thus, confirming whether or not the hydrophobic groups of PS are exposed during cell apoptosis and whether ICG shows affinity for apoptotic cells require newly designed experiments. In this study, we focused specifically on the necrosis avidity of ICG.

The strong avidity of certain small molecular compounds to necrotic tissues has been intensively studied over the last 20 years, mainly by Ni *et al*.^15^. These compounds share a common necrosis-avidity property despite their diverse chemical structures. They generally, but not exclusively, involve certain natural and synthetic chemicals, pigments, dyes, or fluorophores such as porphyrins, hypericin, Evans blue, and Congo red, and function as diagnostic contrast materials for MRI, nuclear scintigraphy, or optical imaging to determine tissue viability, or even as therapeutics or theranostics^15^. Many of these compounds (e.g., porphyrin and hypericin derivatives) were initially developed as “tumour-specific” agents, but were subsequently discovered to be specifically necrosis-avid^43^,^44^. Therefore, it is not surprising for ICG to emerge as another necrosis-avid agent. However, to the best of our knowledge, the necrosis avidity of ICG has not been reported until now, even though it has been applied in clinical practice for more than 40 years. ICG is the only NIR fluorescent dye approved by the FDA for use in clinical practice; thus, the discovery of its necrosis avidity and the potential for immediate direct clinical translation make it superior to other necrosis-avid materials. The possible mechanisms of the necrosis avidity of most of the materials mentioned above are still largely hypothetical^15^. The necrosis avidity has been suggested to be compound specific, and PS, or cholesterol components in the phospholipid bilayer of cellular membranes may be the major targets^10^. The speculation and observational findings of our study are in line with these previously proposed mechanisms^15^. However, a conclusive mechanistic discovery cannot be claimed yet, since the exact binding structure was not identified and we did not perform a competition study. According to our observations, the necrosis avidity of ICG seems to be conditional, because ICG alone did not show necrosis selectivity, and was only evident when bound to LP.

An improved ICG administration strategy was also proposed herein, which could double the SBR under the FDA-approved dose. This strategy significantly enhanced ICG-LP delivery to necrotic lesions and thus offered better sensitivity for necrosis detection. This further suggests that we could extend the potential clinical applications of FMI with ICG for a broad spectrum of necrosis-associated diseases with a common diagnostic dose. After applying FMI to various disease mouse and rat models for necrosis detection, the results confirmed that this optical imaging technique offers excellent boundary delineation of the necrotic area as well as superb sensitivity, especially for the detection of small necrotic lesions (less than 1 mm).

Since NIR FMI has many inherent advantages such as real-time and continuous observations, high sensitivity, high superficial resolution, better tissue penetration (compared with visible light), no ionizing radiation, simplicity, and cost-effectiveness, it appears to be extremely suitable for intraoperative image-guided surgery. Therefore, our discovery also shows the potential of this novel optical technique for the surgical treatment of necrosis-associated disorders. Toward this end, we developed the in house-modified fluorescent microscopy system for the laboratory and preclinical environment. The preclinical applications showed that with the help of real-time image guidance, the system could achieve complete removal of the necrotic tissue with minimum sacrifice of the neighbouring healthy tissue.

The major limitation of this technique is the shallow imaging depth, which is the Achilles heel of many optical imaging techniques for their clinical translation. This problem may lead to false-negative detection of the necrotic lesions deep inside organs, but can be partially compensated for by imaging the surgical field during the operation, since deeper lesions may be exposed on the wound surface. Despite this limitation, there is no doubt that the application of ICG and intraoperative FMI can provide valuable information of necrosis in addition to that obtained with conventional diagnostic imaging scans. Based on our findings, we will enrol more patients with necrosis-associated diseases to test the treatment outcomes in the near future.

Besides our applications in this study, ICG has been applied to many other novel imaging techniques, including fluorescence endoscopic imaging^45^,^46^, fluorescence molecular tomography^47^,^48^, and photoacoustic imaging^49^,^50^, as well as emerging therapeutic methods, including photothermal therapy and photodynamic therapy for anti-tumour and anti-bacterial applications^51^,^52^,^53^,^54^. By integrating these techniques, we believe that our discovery may benefit personalized patient care and contribute to more precise assessments of various necrosis-associated diseases.

## Methods

### Reagents

ICG was purchased from Dandong Medical and Pharmaceutical Co. Ltd. (Dandong, China), which has approval from the Chinese FDA for producing ICG for clinical applications. Foetal bovine serum (FBS) was purchased from Life Technologies Corporation (Beijing, China). PC was purchased from Sigma-Aldrich Co. LLC (Beijing, China).

### Mechanism discovery at the cellular level

The same generation of 4T1-fluc cells (obtained from the Chinese Academy of Military Medical Sciences) was used to compare images of the ICG and ICG + LP groups. Cell necrosis was achieved by removing the culture medium and adding distilled water (5 ml) into the culture dishes for 15 min. Fluorescence microscopy confirmed the swelling, rupture, and necrosis of the cells. ICG was dissolved in PBS and FBS, respectively, with the same concentration (1 μg/ml). Because of such a low concentration, all ICG molecules were bound with LP in FBS^26^. Then, the ICG + PBS and ICG + FBS solutions (5 ml each) were directly added into two dishes of viable cells, respectively. Thirty minutes later, the cells were rinsed with PBS three times to wash out the free ICG or ICG-LP molecules. For the necrotic cells, the cell debris was separated from the supernatant by centrifugation (Multifuge X1 Centrifuge, Thermo Scientific, Waltham, MA, USA; 1500 rpm, 10 min). Then, the ICG + PBS and ICG + FBS solutions (5 ml each) were added into two centrifuge tubes with cell debris, respectively. Thirty minutes later, the cell debris was separated from the supernatant again and rinsed with PBS three times to wash out the free ICG or ICG-LP molecules. All procedures were performed at a constant temperature of 37 °C. Finally, the samples of ICG or ICG-LP with viable and necrotic cells were imaged using our in house-designed fluorescence microscope. The experiment was performed in triplicate.

### Mechanism discovery at the molecular level

ICG was dissolved in PBS and FBS, respectively, with the same concentration (1 μg/ml). The ICG + PBS solution (200 μl) was added into the first column of a 96-well plate as the reference. The ICG + FBS solution (200 μl; containing ICG-LP molecules) was added into the second column. The ICG + FBS solution was mixed with PC, and then 200 μl of the mixture was added into the third column. All procedures were performed at a constant temperature of 37 °C. Fluorescent images of the 96-well plate were acquired using the IVIS Spectrum system (default setting for fluorescence imaging of ICG: excitation 780 nm, emission 831 nm, automatic exposure; Caliper Life Sciences, Hopkinton, MA, USA). The experiment was performed in triplicate.

### Animal experiments

All animals were purchased from the Beijing Vital River Laboratory Animal Technology Co., Ltd. All experimental protocols were approved by the Animal Care and Ethics Committee of Zhujiang Hospital, Southern Medical University, and all the methods were carried out in accordance with the approved guidelines. Animal surgical and imaging procedures were performed under isoflurane gas anaesthesia (3% isoflurane-air mixture). The mice and rats were sacrificed via intraperitoneal injection of 4% chloral hydrate (0.15 ml for mice and 1 ml for rats). All efforts were made to minimize suffering. The Balb/c nude mice were 6–8 weeks old and 20–25 g. Female mice were used for establishing 4T1 breast cancer xenografts, and male mice were applied for the other mouse models. The Sprague-Dawley rats were all male, 3–8 weeks old, and 150–200 g.

### *In vivo* and *ex vivo* verifications

For the burn-induced hind limb necrosis model, six mice were anaesthetized under isoflurane gas, and each received a continuous laser-beam burn (2 W output, 808 nm wavelength, and 8 min irradiation) on the backside of its left hind limb using the MDL-H-808-2W system (Changchun New Industries Optoelectronics Technology, Changchun, China). Twenty-four hours later, all mice received IV injections of ICG (0.1 ml, 0.5 mg/kg) and were imaged with the IVIS system at 5 min, 6 h, 12 h, 18 h, 24 h, 5 days, and 9 days post-injection. The region of interest (ROI) of the necrotic area was manually delineated. The ROI or normal area was defined as the symmetrical body area of the right hind limb. Then, the corresponding optical efficiency and SBR were quantitatively measured and calculated for both the necrotic and normal tissues.

For the muscle ischemia-reperfusion model, six mice were anaesthetized, and the dorsal skin of each left hind limb was surgically opened; a muscle bundle was separated and its ends were ligated using a 1-0 silk thread to block the blood supply. Twelve hours later, the ligation was surgically removed to achieve reperfusion of the blood^34^. Another 12 h later, all mice received IV injection of ICG, following the same protocol for image acquisition and analysis as described above for the burn necrosis mouse model. After the final imaging acquisition on the ninth day, the mice were sacrificed for TTC staining following the standard procedure^35^. We then employed our in-house fluorescence microscope to acquire the fluorescent images for *ex vivo* verification.

### SBR enhancement experiment

Burn necrosis mouse models (n = 12) were established as described above. The control group (n = 6) received an IV bolus injection of ICG (2.0 mg/kg). The experimental group (n = 6) received 4 intermittent injections (0.1 ml each, 0.5 mg/kg) at 3-h time intervals. Fluorescent images were acquired from both groups using the IVIS system at 12 h, 18 h, 24 h, and 30 h post-injection. For the experimental group, the timing started from the last IV injection of ICG. The IV bolus injection and the last intermittent injection were performed at the same time for the two groups.

### Fluorescence imaging using the in-house microscopy system

The fluorescence microscope was coupled with a conventional camera and a low-temperature CCD (PIXIS CCD, Princeton Instruments, Trenton, NJ, USA) to acquire both white-light and fluorescent images. The laser provided 775 ± 25 nm excitation, and the emission was obtained with 845 ± 25 nm filtering. All of the fluorescent images of organs were acquired with an aperture of F1.4 and exposure of 0.1 s, and the fluorescent images of hematoxylin and eosin (H&E)-stained slices were captured with an aperture of F1.4 and exposure of 1.0 s. For the *in vivo* applications of the intraoperative image-guided surgery, the magnification was reset for each image based on the surgeon’s specific request. Since the fields of view of the two cameras were different, we developed an algorithm to achieve automatic imaging registration of the white-light and fluorescent images.

### Necrosis boundary definition and small-lesion detection

A 4T1-fluc xenograft mouse model (n = 6) was established by subcutaneously injecting 2 × 10^6^ 4T1 cells into the upper torso of each female mouse. One week later, bioluminescent images were acquired by the IVIS system to confirm tumour survival using the intraperitoneal injection of luciferase. Two weeks later, the average tumour lesion size reached 12 ± 2.7 mm, and each mouse received an IV injection of ICG (0.1 ml, 0.5 mg/kg). After another 24 h, the mice were sacrificed, and the tumour lesions were resected and split. The white-light and fluorescent images of the tumour specimens were then acquired using our in-house fluorescence microscopy system. The specimens were stained for TTC, and white-light images were taken again using the fluorescence microscope.

Five mice were anaesthetized, and laser irradiation (2 W output, 808 nm wavelength, 8 min) was performed on the skin above the left side of the brain to cause brain necrosis. Twelve hours later, the three surviving mice received an IV injection of ICG (0.1 ml, 0.5 mg/kg). Twenty-four hours after the injection, the mice were sacrificed. The heads were removed, immersed in a tissue-freezing medium (Leica Microsystems Nussloch GmbH, LEICA, Wetzlar, Germany), and frozen at −80 °C. Then, slices were prepared using a cryostat microtome (Leica CM1950, LEICA, Wetzlar, Germany) to obtain coronal planes of the necrotic tissue. The sections were imaged and stained for TTC following the same protocol as described above for the 4T1 tumour experiment.

Laparotomy was performed on the three mice to expose the liver. The blood circulation of a part of the left liver lobe was blocked using a liver occlusion clamp. That part was then injected with 0.1 ml pure ethanol. One minute after the injection, the clamp was removed, and the abdomen was closed with a two-layer suture. ICG (0.1 ml, 0.5 mg/kg) was IV-injected 24 h after the operation. Another 24 h later, the entire liver was excised for white-light and fluorescent imaging, and then sliced for H&E staining and imaged using fluorescence microscopy to verify the boundary of the necrotic tissue.

Six rats were fed with water only 12 h pre-operatively, and the abdominal fur was removed. Laparotomy was performed on each rat to expose the anterior wall of the stomach. Acetic acid (0.01 ml; Beijing Chemical Works, Beijing, China) was injected in the gastric submucosa to create a gastric ulcer^55^. In order to establish a double-blind experiment, the injection site was randomly chosen in the stomach by a surgeon without notifying the imaging operators. The abdomen was closed with a double suture, and ICG was IV-injected (0.3 ml, 0.5 mg/kg) about 72 h later. Another 24 h later, the rats were sacrificed to obtain their stomachs. Each stomach was surgically opened along the greater gastric curvature, and the serosal and mucosal surface was imaged using the fluorescence microscope. After detection of the gastric ulcer, the tissue samples were stained for H&E, and the lesion boundary definition was verified with microscopy.

### Preclinical feasibility of the intraoperative image-guided surgery

*In vivo* escharectomy was performed on three burn necrosis mice, and the in-house fluorescence microscopy system was used to provide intraoperative imaging guidance. ICG was injected 24 h before the surgery. During the surgery, all fluorescent images were acquired with the room light off, but the surgical procedures were performed with the room light on. After anaesthesia, the first set of fluorescent images was acquired to delineate the necrosis boundary, and then a surgeon removed the necrotic tissue. During this procedure, fluorescent images were acquired sporadically based on the surgeon’s request to evaluate the residual tissues until the necrotic tissue was completely removed.

Using a similar procedure, *in vivo* debridement was performed on three bacterial abscess mice with intraoperative imaging guidance using the fluorescence microscopy system. The model was established by subcutaneously injecting 1 × 10^8^ colony forming units of methicillin-resistant *Staphylococcus aureus* (type ST-239) into the abdominal wall of each mouse. The strains were obtained from the Institute of Microbiology of Southern Medical University, following the US Committee for Clinical Laboratory Standards criteria^56^. During the surgery, the fluorescent images were acquired sporadically based on the surgeon’s request to achieve complete removal of pus and necrotic tissues.

### Statistical analyses

Statistical comparisons were made using the Student’s t-test in GraphPad Prism 5 software. SBR values are expressed as mean ± SD. P values less than 0.05 were considered to be statistically significant. Means and SDs were calculated for experiments performed at least three times.

## Additional Information

**How to cite this article**: Fang, C. *et al*. Illuminating necrosis: From mechanistic exploration to preclinical application using fluorescence molecular imaging with indocyanine green. *Sci. Rep*. **6**, 21013; doi: 10.1038/srep21013 (2016).
